# Authorship and Contributorship

**Published:** 2011

**Authors:** Mehran Zarghami

**Affiliations:** 1 Department of Psychiatry and Psychiatry and Behavioral Sciences Research Center, Mazandaran University of Medical Sciences, Sari, Iran.

## Abstract

Authorship which is important to the grant support, academic promotion and reputation of the scientists and their institution should be determined by how genuinely they contribute to each article. The International Committee of Medical Journal Editors (ICMJE) has recommended that authorship credit should be based on substantial contributions to conception and design, acquisition of data, or analysis and interpretation of data; drafting the manuscript or critical revising it; and ultimate approval of the version to be published. Interpretation of the respective contributions of individual authors is not possible from order of authorship. The corresponding author should certify that all authors fulfill standards for authorship and prepare a brief written description of their contribution to the manuscript. This information provides an appropriate context for the readers of the articles to be able to interpret the authors’ roles correctly.

Ethics is generally defined as “doing something right” (1). In other words, “Ethics is a body of principles or standards of human conduct that govern the behavior of individuals and groups.” (1) Ethical behavior is more than a simple belief; it also includes personal, group and organizational act (2). A major part of ethics arises directly or indirectly from divine law. Also a natural body of laws arises from human nature itself (3).

Ethics cover a number of domains such as family, society, politics, medicine, education, and research. Ethical considerations in medical research are different from other domains. A point which may be considered ethical in everyday life may be immoral in the field of research. Doing something instead of someone else may be considered an ethical devotion in many domains of everyday life. However, it is not ethical to express a scientific opinion on behalf of another scientist when s/he has not been informed. So what should be considered ethical by a researcher is different from that of a lay person. This point has not been established completely in emerging research communities yet.

Compliance with ethical issues is essential in all stages of any research. It is therefore important to consider these issues at each stage of research before the final plan.

Academic honesty in all phases of research is the first principle. The main researcher is responsible to consider ethics in research. But research Council or committee of each institution should control and supervise all aspects of research, including ethical considerations. Research Committee may explore ethical issues of research projects directly, or devolve this responsibility to the Advisory Committee (Committee for Ethics in Research), which is selected among experts and researchers.

It is expected that every research committee considers ethical issues in all phases of research, including adoption of research topic, statement of problem, literature review, planning and implementation of research, data gathering, human resources and management, as well as interpretation of clinical findings, statistical analysis, and finally, reporting and publishing it.

The research committee should articulate guidelines defining responsibilities and rights, to provide a climate where members can fulfill their special obligations for effective functioning (3). Some of the guidelines are appropriate to all communities of every university. While other guidelines focus on factors unique to each research community and its peculiar attributions (3).

In recent decades, many universities have sanctioned regulations and policies for ethical considerations in the research conduct, conflict of interest, and reporting of research; guarantorship, authorship and contributorship. The latter is the focus of this editorial.

An author is narrowly defined as “the originator of any written work”. Broadly defined, an author is "the person who originates or gives existence to anything", and generally referred to be someone who has made essential intellectual contributions to a publi-shed study (4), and auth-orship assigns responsibi-lity for what is created and gives credit for intellectual work (5,6).

Biomedical authorship has important academic, social, and financial inferences (4). Authorship which is important to the grant support, academic promotion and reputation of the individuals and their institution should be determined by how genuinely they con-tribute to each article (6). International Committee of Medical Journal Editors (ICMJE) has established standards for authorship, contributorship and guarantorship which are similar on basic issues.

Recently a published material in a web blog indicates that in dissertations by Swedish medical school students, there are names of authors who did not have any substantial intellectual contribution to their studies. Unfortunately, similar findings can be seen in many other countries, including Iran. However, published studies about this issue are sparse (7).

Various reasons and motives interfere with authorship, contributorship and guarantorship standards all over the world. This problem seems to be more in developing countries (6,7). Senior institution and faculty members want to be seen as active investigators even though their other responsibilities prevent them from making direct contributions to research projects (6). Indeed, authorities in medical schools developed their views of authorship, and teach their students indirectly that honorary or guest authorship is acceptable due to their administrative, financial, and logistic support (6,7). Common sense challenges this view. It is not easy to believe that senior scientists and investigators with many years of academic experience do not acknowledge that they don’t have the right to put their names as authors of the articles without significant contribution. They look at guest authorship as an “aspect of seniority”. Some of them consider their junior colleagues as peons. Indeed, this is a vicious cycle; many of them argue that they have been the “victims” of similar misconducts when they were juniors themselves (7). This is an accepted idea in some research centers. In some cases simply proposing a new idea for conducting research fulfills the requirements of being an author (7).

On the other hand, insertion of authorities and senior colleagues as authors will improve the credibility of the manuscripts and its chance for publication (6). Moreover, junior researchers may not want to annoy their chiefs, who hold actual efficient power over research opportunities, and promotion, or even their employment (6). Sometimes breaching the authorship criteria is a way of expressing gratitude of junior researchers to their seniors; I’ll put your name in the byline of my manuscript because you have done me a favor and accepted me as a guest author in your article without significant contribution!

There are many examples of premature scientific promotions in junior scientists who have had no time for making direct contributions to research projects because of their executive responsibilities; some of them have been appointed as if they are legends.

Even in universities and research centers which are sensible to authorship and contributorship, sometimes controversies arise about who the author is, and how the authors should be listed. Although authorship practices differ from one setting to another, implementation of established standards for authorship, contributorship and guarantorship, such as the ICMJE standards may prevent controversies in this field. Alteration in this practice should be within these basic guidelines.

Order of authorship is another heading that has no generally agreed upon meaning, and is determined in different ways across disciplines, research centers, and countries. As a result, interpretation of the respective contributions of individual authors is not possible from order of authorship. Everyone who seeks to understand how an author has contributed to the article should not consider the order of authorship (6). For examples some authors put their names in the byline of the manuscript in descending order of contribution, others place the author who took the lead in doing the research or writing the article first and the most experienced contributor last or vice versa. Alphabetical or random orders are other disciplines which are rarely seen in some journals (6).

The corresponding author should take responsibility for at least one component of the work, must integrate the responsibility of each other author for other components of the work, and ideally be confident in their co-authors’ efficiency, capability and integrity (8). S/he should certify that all authors fulfill standards for authorship and prepare a brief written description of their contribution to the manuscript. Unfortunately, readers are rarely provided with facts about contributions to studies from persons listed as authors and in Acknowledgments (9).

Some scientific journals now request and publish information about the contributions of each person who has participated in a study, at least for original research (8). So readers can interpret the authors’ roles correctly. ICMJE strongly encourages editors to evolve and implement an authorship and contributorship policy, as well as a policy on identifying the corresponding author (8).

While authorship, contributorship and guarantorship policies apparently remove much of the uncertainty and doubtfulness encompassing contributions, they leave unconcluded the question of the quality and quantity of contribution that qualify for authorship (8). The ICJME has recommended that authorship credit should be based on substantial contributions to conception and design, acquisition of data, or analysis and interpretation of data; drafting the manuscript or critical revising it; and ultimate approval of the version to be published. Authors should meet all of these three conditions (8). When a multicenter group has carried out the research, the group should identify the individuals who accept direct responsibility for each part of the work (10). These individuals should completely meet the criteria for authorship/ contributorship. The ICJME believes that general supervision of the research group alone, acquisition of funding, or data collection does not constitute authorship (8).

Some medical journals also request that one or more authors take responsibility for the integrity of the work as a whole, from inception to published article. These journals also publish their names at the end of the article. These persons are called “guarantors” (8).

All persons who contribute to the study and do not meet the criteria for authorship should be listed in the Acknowledgments; including a department chairperson who provided only general support and persons who provided purely technical help or writing assistance (8). The corresponding authors or guarantors should declare whether they had assistance with study design, data collection, data analysis, or manuscript preparation. In these situations, the corresponding author should disclose the identity of the individuals who have assisted or supported the authors. Material and financial support should be acknowledged too (8).

Individuals or groups who have assisted the research group but whose contributions do not justify authorship may be listed under such headings as “participating investigators” or “clinical investigators,” and their contribution should be described—for example, “critically reviewed the study proposal,” “provided and cared for study patients,” “collected data,” or “served as scientific advisors.” These persons must give written permission to be acknowledged, because readers may argue their endorsement of the data and conclusions (8).

Considering the above mentioned issues, Iranian Journal of Psychiatry and Behavioral Sciences (IJPBS) takes the opportunity to initiate the new policy to request the authors to submit and publish information about the contributions of each person who has participated in a study to promote ethical issues., The journal also welcomes the ICMJE suggestions, considers the authorship/ contributorship standards and provides an appropriate context for the readers of the articles to be able to interpret the authors’ roles correctly in this way.

## Notes

**Declaration of Interest:** None.

## Notes

*Doing something instead of someone else may be considered an ethical devotion in many domains of everyday life. However, it is not ethical to express a scientific opinion on behalf of another scientist when s/he has not been informed.* *So* *what* *should be* *considered** ethical by a researcher **is different from that of a lay person. This* *point** has not been established completely **in* *emerging research communities yet.*

*There are many examples of premature scientific promotions in junior scientists who have had no time for making direct contributions to research projects because of their executive responsibilities; some of them have been appointed as if they are legends.*

*General supervision of the research group alone, acquisition of funding, or data collection does not constitute authorship.*
